# EM-DeepSD: A Deep Neural Network Model Based on Cell-Free DNA End-Motif Signal Decomposition for Cancer Diagnosis

**DOI:** 10.3390/diagnostics15091156

**Published:** 2025-05-01

**Authors:** Zhi-Yang Zhao, Chang-Ling Huang, Tong-Min Wang, Shi-Hao Zhou, Lu Pei, Wen-Hui Jia, Wei-Hua Jia

**Affiliations:** 1 School of Public Health, Sun Yat-sen University, Guangzhou 510080, China; zhaozhy63@mail2.sysu.edu.cn (Z.-Y.Z.); huangchling3@mail2.sysu.edu.cn (C.-L.H.); zhoushh33@mail2.sysu.edu.cn (S.-H.Z.); jiawh3@mail2.sysu.edu.cn (W.-H.J.); 2 Sun Yat-sen University Cancer Center, Guangzhou 510060, China; wangtm@sysucc.org.cn (T.-M.W.); peilu@mail2.sysu.edu.cn (L.P.)

**Keywords:** cell-free DNA, cancer diagnosis, signal decomposition, deep neural network

## Abstract

**Background and Objectives:** The accurate discrimination between patients with and without cancer using their cell-free DNA (cfDNA) is crucial for early cancer diagnosis. The end-motifs of cfDNA serve as significant cancer biomarkers, offering compelling prospects for cancer diagnosis. This study proposes EM-DeepSD, a signal decomposition deep learning framework based on cfDNA end-motifs, which is aimed at improving the accuracy of cancer diagnosis and adapting to different sequencing modalities. **Materials and Methods:** This study included 146 patients diagnosed with cancer and 122 non-cancer controls. EM-DeepSD comprises three core modules. Initially, it utilizes a signal decomposition module to decompose and reconstruct the input end-motif profiles, thereby generating multiple regular subsequences that optimize the subsequent modeling process. Subsequently, both a machine learning module and a deep learning module are employed to improve the accuracy of cancer diagnosis. Furthermore, this paper compares the performance of EM-DeepSD with that of existing benchmarked methods to demonstrate its superiority. Based on the EM-DeepSD framework, we developed the EM-DeepSSA model and compared it with two benchmarked methods across different cfDNA sequencing datasets. **Results:** In the internal validation set, EM-DeepSSA outperformed the two benchmark methods for cancer diagnosis (area under the curve (AUC), 0.920; adjusted *p* value < 0.05). Meanwhile, EM-DeepSSA also exhibited the best performance on two independent external testing sets that were subjected to 5-hydroxymethylcytosine sequencing (5hmCS) and broad-range cell-free DNA sequencing (BR-cfDNA-Seq), respectively (test set-1: AUC = 0.933; test set-2: AUC = 0.956; adjusted *p* value < 0.05). **Conclusions:** In summary, we present a new framework which can achieve high classification performance in cancer diagnosis and which is applicable to different sequencing modalities.

## 1. Introduction

Cell-free DNA (cfDNA) represents a complex mixture of fragmented DNA that is derived from diverse tissues through cell necrosis and apoptosis, and which circulates in the blood [1,2]. In recent years, there has been considerable interest in exploring the potential of cfDNA in plasma as a biomarker for cancer diagnosis, particularly regarding its unique fragmentation patterns, which multiple studies have demonstrated to exhibit superior cancer diagnosis performance compared to other cfDNA features such as the copy number variations and fragment length [3,4,5].

Some studies have illuminated that the fragmentation patterns of cfDNA are non-random, with certain regions of the genome being more susceptible to breakage into DNA fragment ends. It has been linked to DNA nuclease activities [1,6]. Serpas et al. found that the frequency of the CCCA end-motif decreased significantly in mice exhibiting a deficiency in deoxyribonuclease 1-like 3 [1]. Jiang and colleagues reported that there was a discrepancy in end-motifs between patients with HCC and those without HCC. They utilized normalized Shannon entropy to devise the Motif Diversity Score (MDS), which is derived from the first four bases at cfDNA’s 5′ end (4-mer EM profile). Notably, the MDS value was markedly elevated in cancer patients [2]. In particular, the 4-mer motifs such as CCCA, CCAG, and CCTG are more prevalent in subjects with HCC versus those without HCC. Other studies have further confirmed the clinical application value of plasma cfDNA EMs for cancer diagnosis [7,8,9,10,11]. Zhou et al. constructed six “founder” end-motif profiles (F-profiles) through non-negative matrix factorization (NMF), and quantified human cfDNA contributions via deconvolution. Encouragingly, the F-profiles showed strong diagnostic potential for hepatocellular carcinoma [12]. Their research also found that F-profiles I, II, and III were linked to deoxyribonuclease 1-like 3 (DNASE1L3), deoxyribonuclease 1 (DNASE1), and DNA fragmentation factor subunit beta (DFFB), respectively. Among the six F-profiles, the use of F-profile I could provide information on human patients with systemic lupus erythematosus. F-profile VI could be used to detect individuals with hepatocellular carcinoma. F-profile VI was more prominent in patients with nasopharyngeal carcinoma who were undergoing chemoradiotherapy.

However, there are still two major deficiencies. Firstly, some methods such as the MDS and F-profiles still have limitations in information extraction, which affects the accuracy of cancer diagnosis using these methods. The MDS primarily accentuated the diversity within the studied EM profiles, and potentially overlooked other significant information. Similarly, F-profiles crafted from mice with diverse nuclease deficiencies excel in related diseases but may encounter constraints in precise cancer diagnostics [13]. Secondly, although directly utilizing EM profiles for machine learning (ML) models is a relatively comprehensive information extraction strategy [9,14], the associated high dimensionality and intricate sequence variations limit their test performance in high-precision cancer prediction [9,15]. Hence, it is imperative to delve into advanced methodologies that can harness intricate information more comprehensively, enhancing the precision of cancer diagnosis.

Signal decomposition techniques can transform a complex sequence into multiple regular subsequences, thereby reducing the difficulty of subsequent modeling [16,17,18,19,20]. Initially, signal decomposition was widely applied in fields like engineering and finance, and was later expanded into medicine, especially disease diagnosis [21,22,23,24,25,26]. Xiao et al. achieved an improved prediction model by decomposing and reconstructing stock prices using singular value decomposition (SSA) [22]. Cura et al. utilized empirical mode decomposition (EMD) and ML to model the electroencephalogram signals of Alzheimer’s patients, and the results showed a superior diagnosis performance [27]. With the development of artificial intelligence technology, machine learning and deep learning techniques such as the least absolute shrinkage and selection operator (LASSO), the elastic net regression algorithm (EN), the random forest (RF) algorithm, extreme gradient boosting (XGBoost), and multilayer perceptron (MLP), attention mechanisms, and long short-term memory (LSTM) have gradually been increasingly applied in the medical field [13,28,29,30,31,32,33]. Mary L. et al. developed a RF model that leveraged methylation profiles and which demonstrated robust performance in the early diagnosis of patients with colorectal, hepatocellular, lung, and gastric cancers [34]. Cristiano’s team employed XGBoost algorithms to model fragmentation patterns of cfDNA, achieving high-precision identification across seven distinct cancer types [35]. Van and colleagues developed the SPOT-MAS model using ML algorithms such as RF, LASSO, EN, and XGBoost, and determined that it can accurately identify five types of cancer: breast cancer, colorectal cancer, gastric cancer, lung cancer, and liver cancer [5]. The attention mechanism method is capable of efficiently capturing key information within data, while LSTM, through its memory cells and gating mechanisms, is able to accurately retain important information while forgetting irrelevant details, which enhances its prediction accuracy [36,37,38,39,40,41]. Shen et al. constructed a language model for cfDNA utilizing attention mechanisms for cancer diagnosis, and the results showed exceptional diagnosis performance [13]. Faiza et al. found that the ADH-Enhancer model, constructed by combining an attention mechanism with LSTM, can accurately identify enhancer sequences [42]. Inspired by the success of the aforementioned cases, we are confident that signal decomposition can be effectively used to decompose EM profiles into regular subsequences, enhancing the resulting information extraction precision with a deep learning framework to achieve high-precision cancer diagnosis.

In summary, we propose an end-motif signal decomposition deep learning framework (EM-DeepSD) that consists of three modules to progressively improve the accuracy of cancer diagnosis. Given the prevalent utilization of whole-genome sequencing (WGS) and whole-genome bisulfite sequencing (WGBS) in plasma cfDNA, we used datasets derived from the two sequencing technologies to develop our model, which was further validated using not only datasets from these two sequencing modalities but also datasets from other sequencing technologies. We benchmarked the diagnosis performance of EM-DeepSD against two methods. EM-DeepSD achieved superior cancer diagnosis performance as compared with the two benchmarked methods. Furthermore, we observed that EM-DeepSD, which was developed using WGS and WGBS datasets, also achieved competitive performance on other data modalities such as 5hmCS and BR-cfDNA-Seq. Therefore, EM-DeepSD has the potential to become a new method for cancer diagnosis.

## 2. Materials and Methods

### 2.1. An Overview of EM-DeepSD

**Figure 1:**
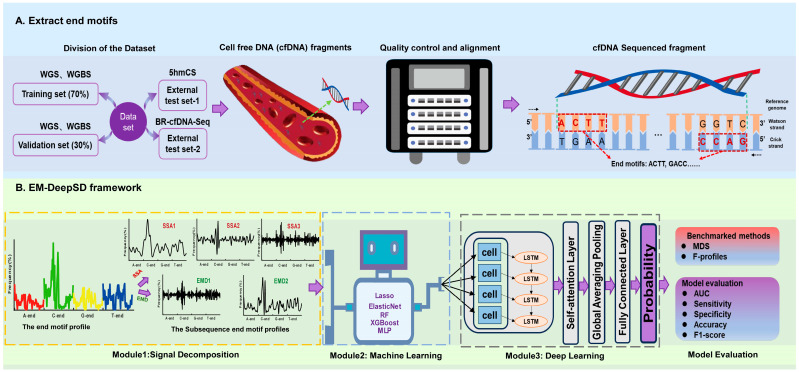
A flowchart depicting the development and evaluation of EM-DeepSD. (**A**) Extraction of end-motifs. (**B**) The architecture of EM-DeepSD, as well as its development and evaluation.

EM-DeepSD is a deep learning architecture comprising three integral modules: a signal decomposition module, a ML module, and a deep learning module. It models EM profiles through these modules, progressively enhancing the precision of cancer diagnosis (Figure 1B). We preprocessed the raw sequencing reads and calculated the frequency of EMs to obtain the input for EM-DeepSD (Figure 1A). The signal decomposition module decomposes and reconstructs the original EM profiles into multiple subsequences with distinct characteristics and more regular structures. This process reduces the complexity of the original data and optimizes subsequent modeling steps. The ML module leverages the strengths of various ML algorithms to extract informative features from subsequences, further enhancing the model’s performance. The deep learning module comprises a LSTM layer, a self-attention layer, a global averaging pooling layer, and a fully connected layer. LSTM is able to accurately retain important information while discarding irrelevant details; therefore, the output of the ML module, which serves as the input, is first conveyed to the LSTM layer. The data processed through the LSTM layer are then passed to the self-attention layer. The self-attention layer dynamically adjusts the importance of elements by computing their interrelationships, thereby efficiently capturing global dependencies and enabling more accurate predictions by considering the influence of other elements when processing each element. To reduce dimensionality and prevent overfitting of the model, the data weighted by the self-attention layer are passed to a global average pooling layer. Finally, the data are passed to a fully connected layer, which outputs prediction probabilities for the identification of cancer. The benchmarked methods include MDS and F-profiles.

### 2.2. Data Collection and Preprocessing

We collected a total of 268 plasma cfDNA samples from across four datasets, covering hepatocellular carcinoma (HCC), head and neck squamous cell carcinoma (HNSCC), colorectal cancer (CRC), lung cancer (LC), nasopharyngeal carcinoma (NPC), breast cancer (BC), pancreatic cancer (PC), glioblastoma (GBM), gastric cancer (GC), and non-cancer control. The sequencing modalities include WGS, WGBS, 5hmCS, and BR-cfDNA-Seq. The datasets derived from WGS and WGBS were partitioned into the training set (N = 129) and validation set (N = 54) through 7:3 stratified random sampling. The training set contains 65 control samples and 64 cancer patient samples, while the validation set comprises 27 controls and 27 cancer patients (Supplementary Table S1).

The WGS dataset (N = 129) consisted of plasma cfDNA samples, which were subjected to WGS, from 34 patients with HCC, 10 patients with CRC, 10 patients with HNSCC, 10 patients with LC, 10 patients with NPC, and 55 controls without cancer. We downloaded it from European Bioinformatics Institute (No.: EGAS00001003409) [2].

The WGBS dataset (N = 54) consisted of plasma cfDNA samples, which were subjected to WGBS, from 17 patients with HCC and 37 controls without cancer. We downloaded it from Genome Sequence Archive (No.: CRA001537) [43].

The 5hmCS dataset (N = 53) consisted of plasma cfDNA samples, which subjected to 5hmCS, from 14 patients with LC, 7 patients with PC, 6 patients with HCC, 4 patients with CRC, 4 patients with GBM, 4 patients with GC, 2 patients with BC, and 12 controls without cancer. We downloaded it from Sequence Read Archive (No.: PRJNA320940) [44].

The BR-cfDNA-Seq dataset (N = 32) consisted of plasma cfDNA samples, which were subjected to BR-cfDNA-Seq, from 14 patients with LC and 18 controls without cancer. We downloaded it from Sequence Read Archive (No.: PRJNA978642) [4].

We conducted quality control on the raw sequencing reads of cfDNA. The sequencing reads were preprocessed by removing the adaptor sequences and low-quality bases (i.e., quality score of <20) [45]. Bowtie2-2.5.2 is used in WGS, 5hmCS, and BR-cfDNA-Seq, while Bismark-0.24.2 is utilized in WGBS, for aligning cfDNA fragments to the reference genome (hg19/GRCh37), with duplicate fragments resulting from PCR being removed [46]. Only paired-end reads that align to the same chromosome with correct orientation and which have an insert size spanning ≤ 600 bp were utilized for downstream analysis [2,47].

We used Bowtie 2-2.5.2 and Bismark-0.24.2 to align the WGS datasets, respectively, and calculated the end-motif frequencies to validate the consistency of different alignment tools in data processing. The results showed that the end-motif frequencies obtained by the two alignment methods were significantly correlated (Supplementary Table S2).

### 2.3. Extract EM Profiles

We referred to the method of Jiang et al. [2] and used Bedtools2-2.31.1 to determine the EMs related to the reference genome at each 5′ fragment end (Watson and Crick strands), with a total of 256 types being obtained. Next, we calculated the frequency of each EM and sorted them according to base sequences to obtain EM profiles for each cfDNA sample.

### 2.4. The Architecture of End-Motif Signal Decomposition Deep Learning Framework (EM-DeepSD)

**Module 1: signal decomposition module.** We used MATLAB (v.R2023a) to perform SSA and EMD for the EM profile of each cfDNA sample.

**(1) SSA.** The processing of SSA is as follows [23,26,48]:

(i) Embedding: In this paper, the original EM profile *X* = (*X*_1_, *X*_2_, …, *X*_N_) was transformed into a matrix of *K* vectors:

(1) $$X=\left[\begin{array}{cccc} X_{1} & X_{2} & \ldots & X_{K}\\ X_{2} & X_{3} & \ldots & X_{K+1}\\ \ldots & \ldots & & \ldots \\ X_{L} & X_{L+1} & \ldots & X_{N} \end{array}\right]$$

where *N* is the number of EMs, which is set to 256. *L* represents the window length (2 ≤ *L* ≤ 2/*N*) [48], and we have set it to 8. The influence of different window lengths on the model performance is discussed in the results.

(ii) Singular value decomposition: Let $S=XX^{T}$, $U_{1},U_{2},\cdots ,U_{L}$ be the eigenvectors of *S*, and $\lambda_{1}\geq \lambda_{2}\geq \cdots \geq \lambda_{L}$ its corresponding eigenvalues. Let $V_{i}=X^{T}U_{i}/\sqrt{\lambda_{i}}$, $U_{i}$ and $V_{i}$ be the left and right singular vectors of matrix *X*, respectively and let $\sqrt{\lambda_{i}}(i=1,2,\cdots L)$ be its corresponding singular values.

(iii) Grouping and diagonal averaging: Based on the contribution rate of singular values, *X* is divided into *m* disjoint subsets ($X=E_{I_{1}}+E_{I_{2}}+\cdots +E_{I_{m}}$). Then, the subsets are reconstructed into *p* subsequences with length *N* through anti-diagonal averaging [23].

**(2) EMD:** EMD was proposed by Huang and colleagues [16]. This method adaptively decomposes the original sequence *X*(*N*) into multiple intrinsic mode functions (IMFs) and a residual based on its characteristics.

(2) $$X(N)=\sum_{m=1}^{M}IMF_{m}(N)+Rse_{M}(N)$$

The process is as follows.

(i) Determine the local maxima and minima points of the original EM profile *X*(*N*). The cubic spline interpolation function is used to construct the upper (*e*(*N*)_(min)_) and lower (*e*(*N*)_(max)_) envelopes, and the mean is calculated.

(3) $$E(N)=\left(e{\left(N\right)}_{\left(\min\right)}+e{\left(N\right)}_{\left(\max\right)}\right)/2$$

(ii) Let *d*_1_(*N*) = *X*(*N*) − *E*(N), and evaluate *d*_1_(*N*) to determine whether it fulfills the criteria for IMFs. If it satisfies the criteria, the first IMF is obtained. If not, repeat the first two steps with *d*_1_(*N*) as the new *X*(*N*). The criteria are as follows: ① the number of zeros and extrema in the sequence are equal or different by at most one; ② the mean of the envelope of local maxima and minima is equal to zero.

(iii) Let *Rse*_1_(*N*) = *X*(*N*) − *d*_1_(*N*). Use *Rse*_1_(*N*) as the new sequence and repeat the first three steps. The EMD process stops when the residual exhibits a monotonic trend or is very small.

(iv) Considering the different number of IMFs among samples and the increased error due to the numerous decomposition layers, permutation entropy (PE) [25] was used to reconstruct new subsequences.

SSA extracted 8 singular values, and reconstructed them into three subsequences based on the singular values’ 1st, 2nd–3rd, and 4th–8th. EMD decomposed these values and reconstructed them into two subsequences based on their PE: high-PE (PE ≥ 0.8) and low-PE (PE < 0.8) (Supplementary Figure S1).

**Module 2: machine learning module.** We constructed ML models, respectively, using sub-sequences obtained through SSA and EMD. The five ML algorithms employed were: LASSO, EN, RF, XGBoost, and MLP. LASSO and EN models are two commonly used linear models; however, when data exhibit intricate nonlinear relationships, traditional linear models often struggle to accurately capture the underlying deep-seated structures [49]. In contrast, nonlinear models such as RF, XGBoost, and MLP demonstrate greater applicability in such scenarios [50,51,52]. In the real world, DNA data are characterized by high dimensionality and a complex structure [39]. Single models focus on different aspects of information extraction and often fail to accurately capture complex data features and, as a result, often fail to meet the requirements for the high-precision diagnostic prediction of cancer [14]. Therefore, we developed a machine learning module that leverages the strengths of multiple ML models, with the objective of achieving greater precision and accuracy in cancer diagnosis.

We utilized the “trainControl” function from the caret package (version 6.0-94) in R (version 4.4.1) to build LASSO, EN, RF, and XGB models. We used the “MLP_Classifier” function from the sklearn (v.0.23.2) software in Python (v.3.10) to build a MLP model. The hyperparameters for the LASSO, EN, RF, XGB, and MLP models were all selected through 10-fold cross-validation. The ML parameters are provided in Supplementary Table S3.

**Module 3: deep learning module.** This module comprises a LSTM layer, a self-attention layer, a global averaging pooling layer, and a fully connected layer, forming a robust deep learning architecture.

**(1) LSTM layer:** The LSTM model, proposed by Schmidhuber et al. [53], benefits from its unique memory cells and gating mechanisms. LSTM is able to accurately retain important information while forgetting irrelevant details, and thereby demonstrates exceptional generalization capabilities. It incorporates a memory cell and three “Gates”: forget gates (*F*), input gates (*I*), and output gates (*O*). The formula is given as:

(4) $$\left\{\begin{array}{l} F_{m}=\sigma (X_{m}W_{xf}+H_{m-1}W_{hf}+b_{f})\\ I_{m}=\sigma (X_{m}W_{xi}+H_{m-1}W_{hi}+b_{i})\\ O_{m}=\sigma (X_{m}W_{xo}+H_{m-1}W_{ho}+b_{o}) \end{array}\right.$$

where *X*_m_ is the output for each ML model, *H* is the hidden state, *W* is the weight parameter, and *b* is the bias parameter.

**(2) Self-attention layer:** The self-attention layer receives data transmitted from the LSTM layer and, through three distinct initial linear transformations, it generates a query (Q) and key (K), along with a value (V). Then, it computes dot-product scores between the Q and the K, scales these scores by dividing by $\sqrt{d_{k}}$, and converts these scaled scores into attention weights via the softmax function. Finally, these attention weights are multiplied by the value (V), yielding the output of the self-attention layer.

(5) $$SelfAttn(Q,K,V)=soft\max(\frac{QK^{T}}{\sqrt{d_{k}}})V$$

**(3) Global averaging pooling layer and fully connected layer:** the global average pooling layer is employed to prevent overfitting of the model, while the fully connected layer uses a sigmoid activation function to introduce nonlinearity and produce the final prediction probability for the identification of cancer.

We trained the module for 50 epochs with an initial learning rate of 0.001 and a batch size of 64. We used Adam as the optimizer. Hyperparameters are key to model performance. To determine the optimal number of hidden layers, we trained models with varying configurations, starting with one layer. We evaluated the performance on the validation set and continued to add layers until the gains were minimal. Similarly, for the number of neurons, we used multiples of 2 (e.g., 4, 6, 8 … 20). Ultimately, we selected a hyperparameter configuration comprising 2 layers with 20 neurons per layer to construct the EM-DeepSSA model; a configuration with 2 layers and 10 neurons per layer was chosen to build the EM-DeepEMD model. After normalization, the deep learning module was trained with Keras (v.2.9.0) of Python (v.3.10) on an Intel I7-12700H CPU with a total of 16 GB of memory.

### 2.5. Motif Diversity Score (MDS)

We calculated the MDS within the EM profiles using the normalized Shannon entropy mathematical equation, based on the formula defined by Jiang and colleagues [2]:

(6) $$MDS=\sum_{i=1}^{256}-P_{i}*\log(P_{i})/\log(256)$$

The theoretical scale ranged from 0 to 1, and *P*_i_ is the frequency of a particular motif.

### 2.6. “Founder” End-Motif Profiles (F-Profiles)

We constructed the EM matrix A, where its rows represent samples and the columns represent 256 EMs. We calculated the percentage contribution (*P_i_*) of each F-profile in a cfDNA sample based on the following formula:

(7) $$A=\sum_{i}\left(P_{i}\times F_{i}\right)$$

where *i* is the index of the F-profile, ranging from 1 to 6, and *F_i_* is the fragmentation profiles given by Zhou and colleagues [12]. *P*_i_ was determined by the non-negative least squares regression (NNLS) using the scipy.optimize.nnls (v.1.5.2) function in Python (v.3.10).

### 2.7. Develop Optimized Motif Diversity Score (MDS-SDs) Based on Signal Decomposition

Based on signal decomposition methods, we developed seven types of optimized motif diversity scores (MDS-SDs) for the diagnosis of cancer. Firstly, for the EM profile of each cfDNA sample, we conducted SSA and EMD, using the procedures that were previously detailed in Module 1. After the signal decomposition, the EMs of the subsequences changed, resulting in some values being less than 0. Therefore, we employed the softmax function for exponential normalization, with values ranging from 0 to 1 and the total sum equaling 1. This enabled us to calculate five types of MDS_Sub_, namely MDS_Sub_-SSA1, MDS_Sub_-SSA2, MDS_Sub_-SSA3, MDS_Sub_-EMD1, and MDS_Sub_-EMD2:

(8) $$\left\{\begin{array}{c} Z_{i}=soft\max(Q_{i})=\exp(Q_{i})/\sum_{i=1}^{256}\exp(Q_{i})\\ MDS_{\mathrm{Sub}}=\sum_{i=1}^{256}-Z_{i}*\log(Z_{i})/\log(256) \end{array}\right.$$

where *Q_i_* is the value of each motif within the subsequences resulting from signal decomposition. Additionally, we constructed comprehensive indices, namely MDS-SSA and MDS-EMD. MDS-SSA was obtained by weighting MDS_Sub_-SSA1, MDS_Sub_-SSA2, and MDS_Sub_-SSA3 using the singular values from the SSA as weights. MDS-EMD was calculated by taking the average of MDS_Sub_-EMD1 and MDS_Sub_-EMD2.

### 2.8. Performance Evaluation Metrics

To assess the classification performance of EM-DeepSD, we chose to calculate five metrics: the area under the ROC (AUC), sensitivity (SEN), specificity (SPE), accuracy (ACC), and F1-score (F1).

(9) $$\left\{\begin{array}{c} SEN=TP/(TP+FN)\\ SPE=TN/(TN+FP)\\ ACC=(TP+TN)/(TP+TN+FP+FN)\\ F1=[(2\times SEN\times TP)/(TP+FP)]/[SEN+TP/(TP+FP)]\\ \mathrm{AUC}:\;\mathrm{Area}\;\mathrm{under}\;\mathrm{the}\;\mathrm{ROC}\;\mathrm{Curve}\; \end{array}\right.$$

where TP and TN, respectively, indicate the number of cancer and non-cancer cases correctly identified by the EM-DeepSD. FP and FN specifically denote the number of cancer and non-cancer cases that could not be correctly identified by the EM-DeepSD. ROC means the receiver operating characteristic curve.

### 2.9. Statistical Analysis

The experiment was conducted using Python (v.3.10), R (v.4.4.1), and MATLAB (v.R2023a). PROC (v.1.18.5) was used to calculate the AUC, sensitivity, specificity, accuracy, and F1-score. DeLong’s test was used to compare the differences between the AUC values, and the *p* values were adjusted using the Benjamini–Hochberg method. This study used the Wilcoxon rank-sum test to compare the differences in EMs, MDS, F-profiles, and MDS-SDs between the cancer and control groups, and the Bonferroni method to adjust the *p* values. A 95% confident interval (95% CI) was presented in a bracket next to each value accordingly. Unless otherwise stated, two-sided tests were employed, and α = 0.05.

## 3. Results

### 3.1. EM Profiles of cfDNA Differences Between Cancer and Control Groups

**Figure 2:**
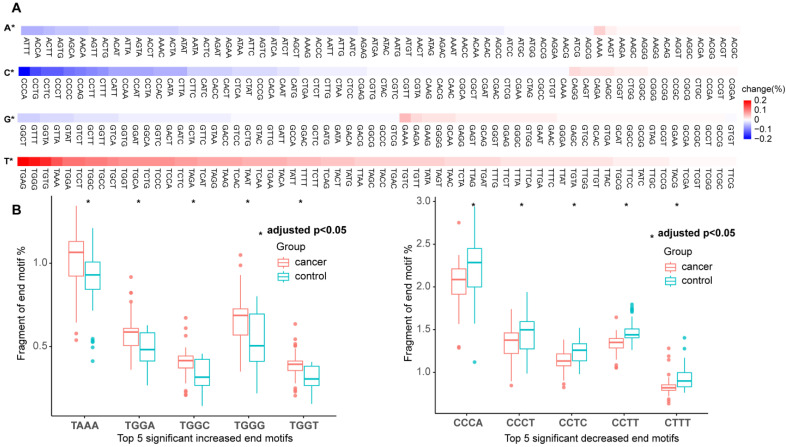
Differences in cfDNA EMs between cancer and control groups. (**A**) Heatmap shows change of 256 EMs in cancer (n = 64) compared to control group (n = 65). A* denotes adenine-EMs; C* denotes cytosine-EMs; G* denotes guanine-EMs; T* denotes thymine-EMs. (**B**) Box plots show the top 10 motifs with significant differences in frequency between cancer (red) and control (green) groups. * Wilcoxon rank-sum test with Bonferroni-adjusted *p* value < 0.05.

In the training set, we compared the differences in the frequencies of EMs between the cancer and control groups. Among the 256 EMs, we detected 41 EMs with significantly increased frequencies and 27 EMs with significantly decreased frequencies (Wilcoxon rank-sum test, adjusted *p* value < 0.05) (Figure 2A). The thymine-EMs were mainly upregulated in the cancer groups, while the cytosine-EMs were mainly downregulated. Figure 2B presents the top 10 EMs that exhibited significant differences. The frequencies of five EMs (TAAA, TGGA, TGGC, TGGG, and TGGT) were significantly increased in the cancer group, while the frequencies of another five EMs (CCCA, CCCT, CCTC, CCTT, and CTTT) were significantly decreased in the cancer group. Therefore, the differences in the frequencies of EMs between the cancer and control groups can serve as potential biomarkers for cancer diagnosis.

### 3.2. Signal Decomposition of EM Profiles and the Construction of MDS-SDs

**Figure 3:**
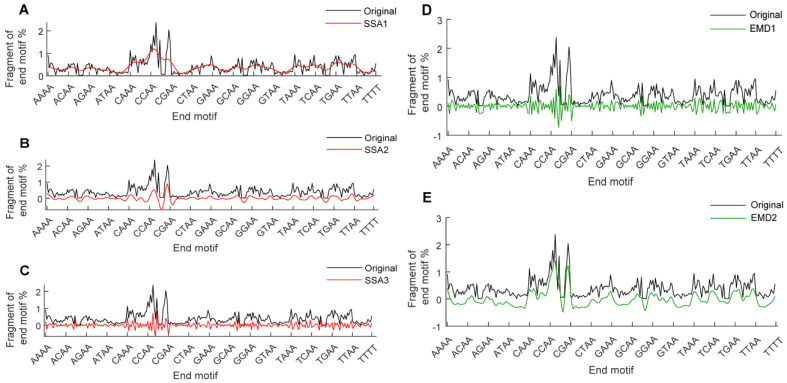
Signal decomposition reconstruction of EM profile for sample AB010. (**A**–**C**) SSA decomposes and reconstructs EM profile into SSA subsequence 1 (**A**), SSA subsequence 2 (**B**), and SSA subsequence 3 (**C**). (**D**,**E**) EMD decomposes and reconstructs EM profile into EMD subsequence 1 (**D**) and EMD subsequence 2 (**E**). Black lines represent original EM profile, red and green lines depict subsequences.

SSA and EMD were performed on the EM profile of each cfDNA sample. Sample AB010, taken as an example, is used to demonstrate the results of the signal decomposition and reconstruction. We found that the subsequences exhibited characteristics that were distinct from the original EM profile and more regular structures. SSA1 and EMD2 portrayed the primary trend of the original profile, reflecting the global characteristics; SSA2 highlighted peaks and troughs, emphasizing local characteristics; SSA3 and EMD1 exhibited jagged fluctuations, and this dense pattern of fluctuations revealed the hidden subtle characteristics within the original profile (Figure 3).

**Figure 4:**
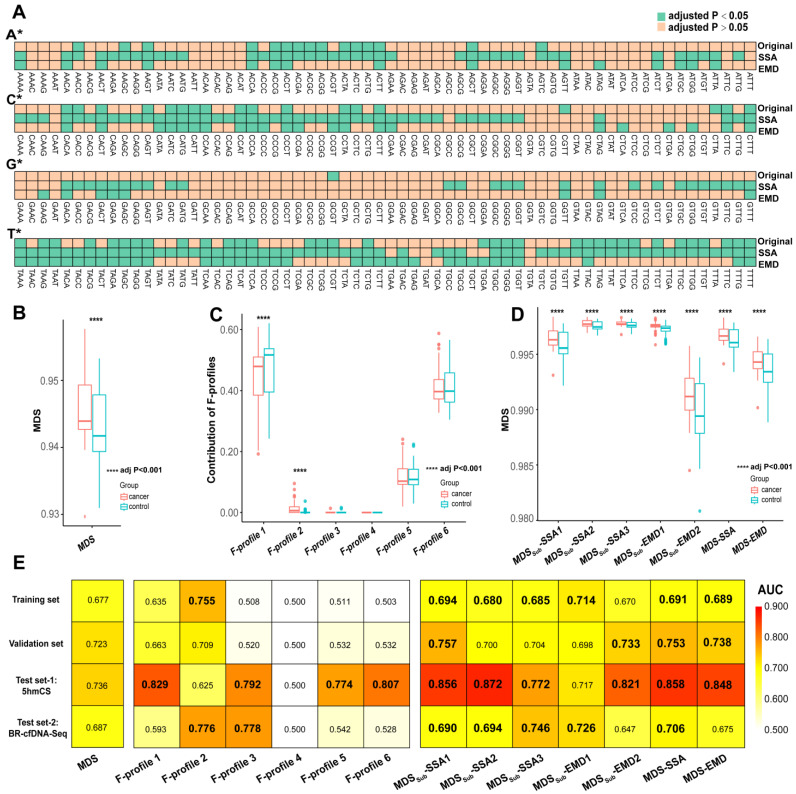
Comparison between original EM profile and subsequences after signal decomposition. (**A**) Heatmap illustrates the differences in the number of significant EMs between original EM profile and subsequences. Green squares represent EMs with significant differences. (**B**–**D**) Box plots show the differences in MDS (**B**), F-profiles (**C**), and MDS-SDs (**D**) between cancer and control groups. (**E**) Heatmap of AUC values for MDS, F-profiles, and MDS-SDs, with AUC values higher than MDS being bolded.

The differences in EMs between the cancer and control groups within the subsequences are presented in Supplementary Figures S2–S4. We observed an increase in T-EMs in SSA1 and EMD2 within the cancer group, which was accompanied by a decrease in C-EMs. The results were consistent with the original EM profiles, which may be attributed to the fact that the two subsequences reflect the fundamental shape of the original profile, i.e., the same global characteristics (Supplementary Figure S2). Among the top 10 motifs with significant differences in SSA2, six motifs did not exhibit significance in the original EMs. The six motifs were located at either the peaks or troughs of the EM spectrum. Specifically, CAGC, CAGG, CCTG, and GTTC were at the peaks, while CCGC and CCGG were at the troughs. The results indicate that SSA2 reflected the local characteristics of the original profile (Supplementary Figure S3). In SSA3 and EMD1, the significant motifs were more scattered and not concentrated around motifs with specific prefixes. This may be because SSA3 and EMD1 capture the subtle characteristics of the original profile (Supplementary Figure S4). Interestingly, the number of significant EMs in the subsequences obviously increased. The original EMs contained only 68 significant EMs, whereas the subsequences based on SSA and EMD, respectively, increased to 169 and 81 (Figure 4A).

We constructed seven optimized MDSs based on signal decomposition, namely MDS-SDs, and calculated both the MDS and F-profiles (see Materials and Methods) of each group. In comparison with the control group, the MDS and MDS-SDs were significantly higher for the cancer group (Wilcoxon rank-sum test, adjusted *p* value < 0.001). However, within the F-profiles, only F-profile 1 and F-profile 2 exhibited significant differences (Figure 4B–D). To evaluate the effect of signal decomposition in improving the accuracy of cancer diagnosis, we calculated the AUC values for the MDS-SDs and two benchmark methods for each dataset. The results show that, across all datasets, the AUC values of most MDS-SDs were higher than that for the MDS (Figure 4E, Supplementary Table S4). In contrast, the performance improvement in the F-profiles was limited to only a few profiles and was not observed in all datasets.

Although the MDS-SDs demonstrated some performance improvements compared to the MDS, the enhancement was not significant. This could be attributed to the fact that the MDS-SDs primarily rely on the diversity of EMs, and fail to fully leverage the advantages of signal decomposition methods and the information within the obtained subsequences.

### 3.3. Machine Learning Based on Signal Decomposition Further Enhances Cancer Diagnosis Accuracy

**Figure 5:**
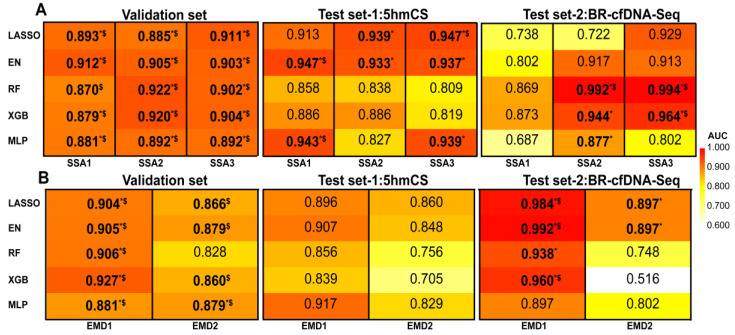
AUC heatmaps show the performances of the ML models. (**A**) AUC heatmaps show the performances of SSA-based ML models on all datasets. (**B**) AUC heatmaps show the performances of EMD-based ML models on all datasets. Using the DeLong test with BH-adjusted *p* value, the AUC of each ML model is compared to the benchmark methods. * Compared to the AUC of MDS, adjusted *p* value < 0.05; ^$^ compared to the AUC of all F-profiles, adjusted *p* value < 0.05. Bold values indicate that the ML model’s AUC is significantly superior to the baseline method.

To further enhance the precision of cancer diagnosis by fully utilizing the information within the obtained subsequences, five ML algorithms were adopted to independently model each subsequence, resulting in a total of 25 ML models (including 15 SSA-based and 10 EMD-based ML models). We compared the performance of the ML models with two benchmark methods and two external test sets for validation. In the first validation set, we found that 20 models achieved significantly higher AUC values as compared with the two benchmark methods (DeLong’ test, BH adjusted *p* value < 0.05). In test set-1, three models achieved significantly higher AUC values as compared with the two benchmark methods (DeLong’ test, BH adjusted *p* value < 0.05). In test set-2, six models achieved significantly higher AUC values as compared with the two benchmark methods (DeLong’ test, BH adjusted *p* value < 0.05) (Figure 5, Supplementary Table S5).

However, the ML models failed to achieve significant enhancements across all datasets simultaneously when compared to the benchmark methods. Despite all ML models performing well in the validation set, their results varied for the external test sets (Figure 5). LASSO-SSA_1–3_, EN-SSA_1–3_, and MLP-SSA_1,3_ exhibited superior performance on test set-1, yet faced limitations on test set-2. Conversely, RF-SSA_2,3_ and XGB-SSA_2,3_ showed excellent performance on test set-2, yet faced limitations on test set-1. The performances of 10 EMD-based ML models were unsatisfactory in test set-1. Therefore, in order to achieve more stable and superior performance, we developed the EM-DeepSD framework.

### 3.4. Development and Validation of EM-DeepSD Framework

**Figure 6:**
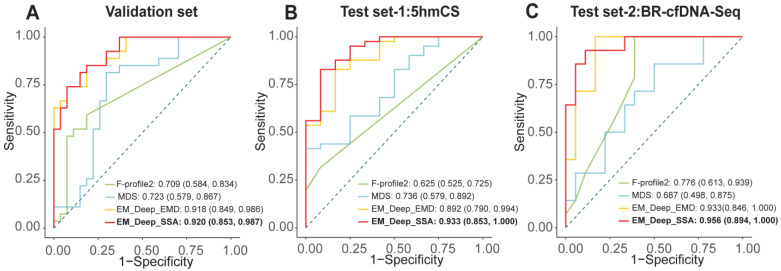
Performance validation of the EM-DeepSD. (**A**–**C**) ROC curves of EM-DeepSD versus benchmarked methods in the validation set (**A**), test set-1 (**B**), test set-2 (**C**).

Based on the EM-DeepSD framework, we developed EM-DeepSSA and EM-DeepEMD, and compared them using the MDS as well as the optimal F-profiles. Among them, EM-DeepSSA exhibited the best performance. Specifically, in the validation set, EM-DeepSSA achieved an AUC of 0.920 (95% CI: 0.853–0.987), outperforming the benchmarked methods (*p* value < 0.05, DeLong’ test, adjusted *p* value < 0.05) while maintaining a high sensitivity of 85.2%, a specificity of 81.5%, an accuracy of 83.3%, and an F1-score of 0.836. Furthermore, in test set-1, subjected to 5hmCS, EM-DeepSSA also demonstrated exceptional performance, achieving an AUC of 0.933 (95% CI: 0.853–1.000) and outperforming the benchmarked methods (*p* value < 0.05, DeLong’ test, adjusted *p* value < 0.05) with a sensitivity of 82.9%, a specificity of 91.7%, an accuracy of 84.9%, and an F1-score of 0.895. In test set-2, subjected to BR-cfDNA-Seq, despite a slightly low specificity of 66.7%, EM-DeepSSA still maintained a high AUC of 0.956 (95% CI: 0.894–1.000) and outperformed the benchmarked methods (*p* value< 0.05, DeLong’ test, adjusted *p* value < 0.05) with a sensitivity of 100.0%, an accuracy of 81.3%, and an F1-score of 0.813 (Figure 6A–C, Table 1). These findings validate the robust and superior performance of EM-DeepSSA across diverse testing scenarios.

We stratified test set-1 based on the cancer types and categorized the cancer types with sample sizes smaller than 10 as other cancer. Meanwhile, we calculated the AUC values and their confidence intervals for lung cancer and other cancers through 1000 Bootstrap iterations. We found that EM-DeepSSA performed the most accurately in predicting other cancers (AUC = 0.957, 95% CI: 0.874–1.000), followed by lung cancer (AUC = 0.884, 95% CI: 0.729–0.987) (Supplementary Table S6).

### 3.5. Ablation Testing of EM-DeepSSA Model and the Impact of SSA Window Length on Cancer Assessment

EM-DeepSSA consists of three modules; thus, we conducted ablation tests by removing the attention mechanism, Module 1, and Module 2, separately. After removing the attention mechanism, the performance achieved by EM-DeepSSA in the diagnosis of cancer was minimally affected. On the validation set and the two external test datasets, the AUC values achieved by EM-DeepSSA were reduced from 0.920 (0.853, 0.987) to 0.914 (0.841, 0.986), from 0.933 (0.853, 1.000) to 0.929 (0.845, 1.000), and from 0.956 (0.894, 1.000) to 0.917 (0.805, 1.000) in the diagnosis of cancer, respectively. After removing both the attention mechanism and Module 1, the AUC values of EM-DeepSSA decreased to 0.907 (0.824, 0.989), 0.915 (0.835, 0.994), and 0.861 (0.730, 0.992) on the three datasets, respectively. The further removal of Module 2, in addition to the attention mechanism and Module 1, resulted in AUC reductions to 0.816 (0.699, 0.934), 0.915 (0.816, 1.000), and 0.675 (0.480, 0.870) on the same datasets. The classification metrics for these ablation test results are provided in Supplementary Table S7.

Furthermore, we systematically investigated the influence of different window lengths for SSA on the EM-DeepSSA models by setting the window lengths to 8, 16, 32, and 128, respectively. The results indicate that the EM-DeepSSA models, with varying window lengths, exhibited outstanding stability and predictive capability on the validation set, with the AUC values being stably distributed between 0.888 and 0.920. Furthermore, in test set-1, the AUC values of the models ranged from 0.904 to 0.943, and in test set-2, they ranged from 0.857 to 0.956 (Supplementary Table S8). These results confirm the robustness and adaptability of EM-DeepSSA.

### 3.6. Influence of Clinical Features on Model Prediction

We stratified the test sets by cancer stage. In test set-1, the model achieved the highest accuracy for stage II, with an AUC of 0.958 (95% CI: 0.871–1.000), while it achieved the lowest for stage I cancers, with an AUC of 0.854 (95% CI: 0.689–1.000) (Supplemental Figure S5A). In test set-2, the AUC for cancers at stage III and above was 0.956 (95% CI: 0.894–1.000) (Supplemental Figure S5B). These results demonstrate that EM-DeepSSA can detect cancers at all stages within the test sets, despite exhibiting a slightly lower performance in the early stages (I) compared to those at stage II and III.

Furthermore, upon stratifying the test sets by gender, we observed that, while the AUC for males was slightly higher than that for females, the difference was not statistically significant (DeLong’ test, test set-1 *p* value = 0.245, test set-2 *p* value = 0.201), indicating that the influence of gender on the model’s predictive ability was limited (Supplemental Figure S5C,D). Next, we evaluated the potential confounding effect of age on our model. By examining the correlation between the model prediction scores and the participants’ ages, we found no significant correlation (Pearson’s correlation, test set-1 R = 0.179, *p* value = 0.200, test set-2 R = 0.242, *p* value = 0.183), indicating that age differences are unlikely to affect the accuracy of our model.

## 4. Discussion

### 4.1. Challenges in Cancer Diagnosis Using the EM Profiles of cfDNA

In an era marked by a global rise in cancer-related morbidity and mortality, precise screening for cancer has emerged as an economical and efficient strategy to alleviate the burden of cancer [54]. As the technology of cfDNA liquid biopsy progresses, EM profiles have shown immense potential in the non-invasive and precise diagnosis of cancer [7,8,15,29,55]. However, there are still two major deficiencies: Firstly, some methods such as MDS and F-profiles still have limitations in extracting information, making it difficult to achieve precise cancer diagnosis. Secondly, due to the high dimensionality and complexity of EM profiles, direct modeling suffers from insufficient performance across different datasets [5,9]. To tackle these issues, we introduce EM-DeepSD, an end-motif signal decomposition deep learning framework.

### 4.2. EM-DeepSD Is Capable of Enhancing the Accuracy of Cancer Diagnosis

Firstly, we developed Module 1, which utilizes SSA and EMD to decompose and reconstruct EM profiles into multiple subsequences with distinct characteristics and more regular structures. This result ties well with those of previous studies wherein electroencephalogram and electrocardiogram signal decomposition were used [24,27], thereby validating the effectiveness of signal decomposition in the context of EM profiles. Next, we constructed seven optimized MDSs (MDS-SDs) based on signal decomposition modules Compared to the MDSs, most MDS-SDs showed superior diagnosis performance. This result may be due to the fact that, after signal decomposition, the differences between the cancer and the control groups are more pronounced in subsequences, and the MDS-SDs obtained through normalized Shannon entropy can detect cancer more accurately.

To further enhance the accuracy of cancer diagnosis, we developed ML Module 2. Our results showed that, despite the potential of ML models to enhance accuracy of cancer diagnosis, none of these models have achieved significant improvements for both benchmark methods across all datasets simultaneously. These results are consistent with the findings of Hou et al. [10], demonstrating the challenges in direct modeling across diverse testing environments and emphasizing the critical importance of enhancing models’ performance. Therefore, to fully leverage the flexibility of various models, we developed the EM-DeepSD framework. Our results showed that EM-DeepSSA outperforms the mainstream state-of-the-art benchmarked methods for cancer diagnosis across different sequencing technologies. This can be attributed to two primary factors. Firstly, our model is derived from cfDNA EM profiles, and previous studies have shown that EMs from different sequencing technologies still exhibit a high degree of correlation [2,5]. Secondly, signal decomposition methods effectively amplify the distinctive characteristics of EM profiles, while the EM-DeepSD framework can extract the strengths of multiple ML models, which enhances its accuracy.

### 4.3. Advantages, Limitations and Future Directions

This study has several advantages. It pioneers a new direction in using cfDNA EM profiles for cancer diagnosis. We successfully applied the signal decomposition method to EM profiles, extracting subsequences that not only reflected distinct characteristics of the original sequences but that also exhibited more regular patterns, enhancing the accuracy of subsequent modeling. Secondly, the EM-DeepSSA model has also demonstrated a certain degree of applicability across different sequencing technologies. Furthermore, based on the above success, we believe that the new framework proposed in this study also has the potential to be applied to other cfDNA features, such as copy number variations and fragment lengths, to achieve more precise cancer diagnosis.

There are several limitations in our study. First, despite using a total of 268 samples from four datasets, the amount of data is still quite small as compared with other cfDNA-based cancer diagnosis models such as SPOT-MAS and ACID [5,13]. Furthermore, due to the limited number of cases for each cancer type, the generalization ability of the model in evaluating “multi-cancer” detection is constrained. Second, this study used datasets from WGS, WGBS, 5hmCS, and BR-cfDNA-Seq sequencing. However, the sample size for each sequencing modality is small, and the performance of EM-DeepSSA on other sequencing modalities remains to be validated. Third, given the evidence suggesting the superiority of EM profiles over other cfDNA features [2,5,15,55], this study has temporarily focused solely on EM profiles. Fourthly, this study only employed two signal decomposition methods, five ML models, and one deep learning model to construct the EM-DeepSD model. The advantages and disadvantages of the EM-DeepSSA model, as compared to models based on other signal decomposition and machine learning methods, remain to be verified.

Therefore, we plan to carry out various forms of cell-free DNA (cfDNA) sequencing on the large number of collected plasma samples, which encompass different cancer types, integrate multiple cfDNA features, and adopt a new approach to achieve the early diagnosis of multiple cancers. In the future, we will conduct a comparative analysis between the EM-DeepSSA model and other relevant models.

### 4.4. Implications for Clinical Practice

The accurate discrimination between patients with and without cancer based on their cfDNA is crucial for early cancer diagnosis. The innovative achievements of this study provide optimization strategies for non-invasive cancer diagnosis technologies. The EM-DeepSD model developed in this study achieves precise cancer identification by integrating decomposition features with deep learning algorithms, and demonstrates applicability across different sequencing scenarios, suggesting potential to promote the implementation of non-invasive cancer screening. However, due to the limited number of cases per cancer type that were used in this study, the model’s generalization capability in multi-cancer detection evaluation remains constrained.

Hence, while the EM-DeepSD model showed promise in cancer diagnosis, further improvements in data quality, feature representation, and model architecture are necessary to fully realize the potential of deep learning in this domain. Future research should focus on addressing these limitations while exploring the integration of large-scale multi-cancer data with advanced deep learning techniques to enhance the diagnostic accuracy and clinical utility of this method.

## 5. Conclusions

In summary, we have creatively proposed a new framework that can gradually enhance the classification performance of cancer diagnosis. The framework comprises three core modules and ingeniously integrates signal decomposition methods with cfDNA EM profiles, and, based on this, we have developed EM-DeepSSA for cancer diagnosis. Our work not only shows the powerful potential of the new framework in the field of fragmentomics, but also introduces innovative ideas for cancer diagnosis, which are expected to facilitate translational research of liquid-biopsy-based early cancer diagnosis in the foreseeable future.

## Figures and Tables

**Table 1:** Classification metrics of EM-DeepSD and baseline methods in diagnosis of cancer.

Data Set	Method	Sensitivity	Specificity	Accuracy	F1-Score
Validation set					
	MDS	0.741 (0.537, 0.889)	0.704 (0.498, 0.862)	0.722 (0.581, 0.831)	0.727
	F-profile 2	0.593 (0.388, 0.776)	0.815 (0.619, 0.937)	0.704 (0.562, 0.816)	0.667
	EM_Deep_EMD	0.852 (0.662, 0.958)	0.815 (0.619, 0.937)	0.833 (0.702, 0.916)	0.836
	EM_Deep_SSA	**0.852 (0.663, 0.958)**	**0.815 (0.619, 0.937)**	**0.833 (0.702, 0.916)**	**0.836**
Test set-1					
	MDS	**0.976 (0.871, 0.999)**	0.250 (0.054, 0.572)	0.811 (0.676, 0.901)	0.889
	F-profile 2	0.317 (0.181, 0.481)	0.917 (0.615, 0.998)	0.453 (0.318, 0.595)	0.473
	EM_Deep_EMD	0.805 (0.651, 0.912)	0.833 (0.516, 0.979)	0.811 (0.676, 0.901)	0.868
	EM_Deep_SSA	0.829 (0.679, 0.928)	**0.917 (0.615, 0.998)**	**0.849 (0.719, 0.928)**	**0.895**
Test set-2					
	MDS	1.000 (0.768, 1.000)	0.000 (0.000, 0.185)	0.438 (0.268, 0.621)	0.609
	F-profile 2	1.000 (0.768, 1.000)	0.500 (0.260, 0.740)	0.719 (0.530, 0.856)	0.757
	EM_Deep_EMD	1.000 (0.768, 1.000)	0.556 (0.308, 0.785)	0.750 (0.562, 0.879)	0.778
	EM_Deep_SSA	**1.000 (0.768, 1.000)**	**0.667 (0.410, 0.867)**	**0.813 (0.630, 0.921)**	**0.813**

Bold values represent the highest-performing method for each metric.

## Data Availability

Data is publicly available at European Bioinformatics Institute (No.: EGAS00001003409), Genome Sequence Archive (No.: CRA001537) and Sequence Read Archive (No.: PRJNA320940 and PRJNA978642).

## Supplementary Materials

The following supporting information can be downloaded at: https://www.mdpi.com/article/10.3390/diagnostics15091156/s1, Figure S1: Boxplots show the singular value contribution rate of SSA and the Permutation Entropy of EMD for the EMs profile of 268 cfDNA samples. Figure S2: Differences in EMs between cancer and control group cfDNA for SSA1 and EMD2. Figure S3: Differences in EMs between cancer and control group cfDNA for SSA2. Figure S4: Differences in EMs between cancer and control group cfDNA for SSA3 and EMD1. Figure S5: The effects of cancer stages and sex on model performance. Table S1: Detailed information of all datasets. Table S2: Correlation analysis of EMs after alignment with Bowtie2 and Bismark in the WGS dataset. Table S3: 10-fold cross-validation for model hyperparameters selection. Table S4: Classification metrics of MDS-SDs and baseline methods in diagnosis of cancer. Table S5: Classification metrics of ML models in diagnosis of cancer. Table S6: In Test Set-1, the AUC values of EM-DeepSSA across different cancer types based on 1000 Bootstrap iterations. Table S7: Classification metrics for the ablation study of EM-DeepSSA. Table S8: Classification metrics for the varying window lengths of EM-DeepSSA. Table S9: Overview of liquid biopsy assays for cancer early detection described in recent publications. Table S10: Summary table of clinical information. Table S11: literature summary table.

## Abbreviations

4-mer EMs profile	the first 4 bases at cfDNA’s 5′ end
5hmCS	5-hydroxymethylcytosine sequencing
95% CI	95% confident interval
ACC	accuracy
AUC	Area Under Curve
BC	breast cancer
BH	Benjamini-Hochberg
BR-cfDNA-Seq	Broad-range cell-free DNA sequencing
cfDNA	cell-free DNA
CRC	colorectal cancer
EMD	Empirical Mode Decomposition
EM-DeepSD	end-motif signal decomposition deep learning framework
EN	Elastic Net Regression
F1	F1-score
GBM	glioblastoma
GC	Gastric cancer
HCC	hepatocellular carcinoma
HNSCC	head and neck squamous cell carcinoma
LASSO	Least Absolute Shrinkage and Selection Operator
LC	lung cancer
LSTM	Long Short-Term Memory
MDS	Motif Diversity Score
ML	machine learning
MLP	Multilayer Perceptron
NPC	nasopharyngeal carcinoma
PC	pancreatic cancer
PE	Permutation Entropy
RF	Random Forest
SEN	sensitivity
SPE	specificity
SSA	Singular Value Decomposition
WGBS	Whole Genome Bisulfite Sequencing
WGS	Whole Genome Sequencing
XGBoost	eXtreme Gradient Boosting

## References

[1] Serpas L., Chan R.W.Y., Jiang P., Ni M., Sun K., Rashidfarrokhi A., Soni C., Sisirak V., Lee W.S., Cheng S.H. (2019). Dnase1l3 deletion causes aberrations in length and end-motif frequencies in plasma DNA. Proc. Natl. Acad. Sci. USA.

[2] Jiang P., Sun K., Peng W., Cheng S.H., Ni M., Yeung P.C., Heung M.M.S., Xie T., Shang H., Zhou Z. (2020). Plasma DNA End-Motif Profiling as a Fragmentomic Marker in Cancer, Pregnancy, and Transplantation. Cancer Discov..

[3] Zhou Q., Kang G., Jiang P., Qiao R., Lam W.K.J., Yu S.C.Y., Ma M.L., Ji L., Cheng S.H., Gai W. (2022). Epigenetic analysis of cell-free DNA by fragmentomic profiling. Proc. Natl. Acad. Sci. USA.

[4] Cheng J., Swarup N., Li F., Kordi M., Lin C.C., Yang S.C., Huang W.L., Aziz M., Kim Y., Chia D. (2023). Distinct Features of Plasma Ultrashort Single-Stranded Cell-Free DNA as Biomarkers for Lung Cancer Detection. Clin. Chem..

[5] Nguyen V.T.C., Nguyen T.H., Doan N.N.T., Pham T.M.Q., Nguyen G.T.H., Nguyen T.D., Tran T.T.T., Vo D.L., Phan T.H., Jasmine T.X. (2023). Multimodal analysis of methylomics and fragmentomics in plasma cell-free DNA for multi-cancer early detection and localization. Elife.

[6] Chen M., Chan R.W.Y., Cheung P.P.H., Ni M., Wong D.K.L., Zhou Z., Ma M.L., Huang L., Xu X., Lee W.S. (2022). Fragmentomics of urinary cell-free DNA in nuclease knockout mouse models. PLoS Genet..

[7] Qi T., Pan M., Shi H., Wang L., Bai Y., Ge Q. (2023). Cell-Free DNA Fragmentomics: The Novel Promising Biomarker. Int. J. Mol. Sci..

[8] Wang Y., Fan X., Bao H., Xia F., Wan J., Shen L., Wang Y., Zhang H., Wei Y., Wu X. (2023). Utility of Circulating Free DNA Fragmentomics in the Prediction of Pathological Response after Neoadjuvant Chemoradiotherapy in Locally Advanced Rectal Cancer. Clin. Chem..

[9] Cao Y., Wang N., Wu X., Tang W., Bao H., Si C., Shao P., Li D., Zhou X., Zhu D. (2024). Multi-Dimensional Fragmentomics Enables Early and Accurate Detection of Colorectal Cancer. Cancer Res..

[10] Hou Y., Meng X.Y., Zhou X. (2024). Systematically Evaluating Cell-Free DNA Fragmentation Patterns for Cancer Diagnosis and Enhanced Cancer Detection via Integrating Multiple Fragmentation Patterns. Adv. Sci..

[11] Jiao Z., Zhang X., Xuan Y., Shi X., Zhang Z., Yu A., Li N., Yang S., He X., Zhao G. (2024). Leveraging cfDNA fragmentomic features in a stacked ensemble model for early detection of esophageal squamous cell carcinoma. Cell Rep. Med..

[12] Zhou Z., Ma M.L., Chan R.W.Y., Lam W.K.J., Peng W., Gai W., Hu X., Ding S.C., Ji L., Zhou Q. (2023). Fragmentation landscape of cell-free DNA revealed by deconvolutional analysis of end motifs. Proc. Natl. Acad. Sci. USA.

[13] Shen H., Liu J., Chen K., Li X. (2024). Language model enables end-to-end accurate detection of cancer from cell-free DNA. Brief. Bioinform..

[14] Hibon M., Evgeniou T. (2005). To combine or not to combine: Selecting among forecasts and their combinations. Int. J. Forecast..

[15] Sundby R.T., Szymanski J.J., Pan A., Jones P.A., Mahmood S.Z., Reid O.H., Srihari D., Armstrong A.E., Chamberlain S., Burgic S. (2024). Early detection of malignant and pre-malignant peripheral nerve tumors using cell-free DNA fragmentomics. Clin. Cancer Res..

[16] Huang N.E., Shen Z., Long S.R., Wu M.C., Shih H.H., Zheng Q., Yen N.-C., Tung C.C., Liu H.H. (1998). The empirical mode decomposition and the Hilbert spectrum for nonlinear and non-stationary time series analysis. Proc. R. Soc. Lond. Ser. A Math. Phys. Eng. Sci..

[17] Ghofrani Jahromi M., Parsaei H., Zamani A., Dehbozorgi M. (2017). Comparative Analysis of Wavelet-based Feature Extraction for Intramuscular EMG Signal Decomposition. J. Biomed. Phys. Eng..

[18] Yang X., Sun J., Yang H., Guo T., Pan J., Wang W. (2025). The heart sound classification of congenital heart disease by using median EEMD-Hurst and threshold denoising method. Med. Biol. Eng. Comput..

[19] Dhongade D., Captain K., Dahiya S. (2025). EEG-based schizophrenia detection: Integrating discrete wavelet transform and deep learning. Cogn. Neurodyn..

[20] Leng W., Yang C., Kou M., Zhang K., Liu X. (2025). Prediction of Patient Visits for Skin Diseases through Enhanced Evolutionary Computation and Ensemble Learning. J. Med. Syst..

[21] Parsaei H., Stashuk D.W., Rasheed S., Farkas C., Hamilton-Wright A. (2010). Intramuscular EMG signal decomposition. Crit. Rev. Biomed. Eng..

[22] Xiao J., Zhu X., Huang C., Yang X., Wen F., Zhong M. (2019). A New Approach for Stock Price Analysis and Prediction Based on SSA and SVM. Int. J. Inf. Technol. Decis. Mak..

[23] Kalantari M. (2021). Forecasting COVID-19 pandemic using optimal singular spectrum analysis. Chaos Solitons Fractals.

[24] Kumar A., Tomar H., Mehla V.K., Komaragiri R., Kumar M. (2021). Stationary wavelet transform based ECG signal denoising method. ISA Trans..

[25] Quinn A.J., Lopes-Dos-Santos V., Dupret D., Nobre A.C., Woolrich M.W. (2021). EMD: Empirical Mode Decomposition and Hilbert-Huang Spectral Analyses in Python. J. Open Source Softw..

[26] Huang Y., Tong S., Tong Z., Cong F. (2021). Signal Identification of Gear Vibration in Engine-Gearbox Systems Based on Auto-Regression and Optimized Resonance-Based Signal Sparse Decomposition. Sensors.

[27] Cura O.K., Akan A., Yilmaz G.C., Ture H.S. (2022). Detection of Alzheimer’s Dementia by Using Signal Decomposition and Machine Learning Methods. Int. J. Neural Syst..

[28] Munguía-Siu A., Vergara I., Espinoza-Rodríguez J.H. (2024). The Use of Hybrid CNN-RNN Deep Learning Models to Discriminate Tumor Tissue in Dynamic Breast Thermography. J. Imaging.

[29] Shen H., Yang M., Liu J., Chen K., Li X. (2024). Development of a deep learning model for cancer diagnosis by inspecting cell-free DNA end-motifs. NPJ Precis. Oncol..

[30] Kwiecinski J., Grodecki K., Pieszko K., Dabrowski M., Chmielak Z., Wojakowski W., Niemierko J., Fijalkowska J., Jagielak D., Ruile P. (2025). Preprocedural CT angiography and machine learning for mortality prediction after transcatheter aortic valve replacement. Prog. Cardiovasc. Dis..

[31] Xu Y.W., Peng Y.H., Liu C.T., Chen H., Chu L.Y., Chen H.L., Wu Z.Y., Wei W.Q., Xu L.Y., Wu F.C. (2025). Machine learning technique-based four-autoantibody test for early detection of esophageal squamous cell carcinoma: A multicenter, retrospective study with a nested case-control study. BMC Med..

[32] Liu T., Guo H., Li Q., Chen K., Xu J., Ma Y., Lin Z., Zhou X., Chen B. (2025). Machine Learning-Enhanced Cerebrospinal Fluid N-Glycome for the Diagnosis and Prognosis of Primary Central Nervous System Lymphoma. J. Proteome Res..

[33] Feher B., de Souza Oliveira E.H., Mendes Duarte P., Werdich A.A., Giannobile W.V., Feres M. (2025). Machine learning-assisted prediction of clinical responses to periodontal treatment. J. Periodontol..

[34] Stackpole M.L., Zeng W., Li S., Liu C.C., Zhou Y., He S., Yeh A., Wang Z., Sun F., Li Q. (2022). Cost-effective methylome sequencing of cell-free DNA for accurately detecting and locating cancer. Nat. Commun..

[35] Cristiano S., Leal A., Phallen J., Fiksel J., Adleff V., Bruhm D.C., Jensen S., Medina J.E., Hruban C., White J.R. (2019). Genome-wide cell-free DNA fragmentation in patients with cancer. Nature.

[36] Zhou S., Xie Y., Feng X., Li Y., Shen L., Chen Y. (2025). Artificial intelligence in gastrointestinal cancer research: Image learning advances and applications. Cancer Lett..

[37] Liu J., Shen H., Chen K., Li X. (2024). Large language model produces high accurate diagnosis of cancer from end-motif profiles of cell-free DNA. Brief. Bioinform..

[38] Hu X., Shi Y., Cheng S.H., Huang Z., Zhou Z., Shi X., Zhang Y., Liu J., Ma M.L., Ding S.C. (2025). Transformer-based deep learning for accurate detection of multiple base modifications using single molecule real-time sequencing. Commun. Biol..

[39] Lee T.R., Ahn J.M., Lee J., Kim D., Park J., Jeong B.H., Oh D., Kim S.M., Jung G.C., Choi B.H. (2025). Integrating Plasma Cell-Free DNA Fragment End Motif and Size with Genomic Features Enables Lung Cancer Detection. Cancer Res..

[40] Zhu G., Rahman C.R., Getty V., Odinokov D., Baruah P., Carrié H., Lim A.J., Guo Y.A., Poh Z.W., Sim N.L. (2025). A deep-learning model for quantifying circulating tumour DNA from the density distribution of DNA-fragment lengths. Nat. Biomed. Eng..

[41] Michel M., Heidary M., Mechri A., Da Silva K., Gorse M., Dixon V., von Grafenstein K., Bianchi C., Hego C., Rampanou A. (2025). Noninvasive Multicancer Detection Using DNA Hypomethylation of LINE-1 Retrotransposons. Clin. Cancer Res..

[42] Mehmood F., Arshad S., Shoaib M. (2024). ADH-Enhancer: An attention-based deep hybrid framework for enhancer identification and strength prediction. Brief. Bioinform..

[43] Zhang H., Dong P., Guo S., Tao C., Chen W., Zhao W., Wang J., Cheung R., Villanueva A., Fan J. (2020). Hypomethylation in HBV integration regions aids non-invasive surveillance to hepatocellular carcinoma by low-pass genome-wide bisulfite sequencing. BMC Med..

[44] Song C.X., Yin S., Ma L., Wheeler A., Chen Y., Zhang Y., Liu B., Xiong J., Zhang W., Hu J. (2017). 5-Hydroxymethylcytosine signatures in cell-free DNA provide information about tumor types and stages. Cell Res..

[45] Chen S., Zhou Y., Chen Y., Gu J. (2018). fastp: An ultra-fast all-in-one FASTQ preprocessor. Bioinformatics.

[46] Krueger F., Andrews S.R. (2011). Bismark: A flexible aligner and methylation caller for Bisulfite-Seq applications. Bioinformatics.

[47] Chan K.C., Jiang P., Chan C.W., Sun K., Wong J., Hui E.P., Chan S.L., Chan W.C., Hui D.S., Ng S.S. (2013). Noninvasive detection of cancer-associated genome-wide hypomethylation and copy number aberrations by plasma DNA bisulfite sequencing. Proc. Natl. Acad. Sci. USA.

[48] Mukhopadhyay S.K., Krishnan S. (2020). A singular spectrum analysis-based model-free electrocardiogram denoising technique. Comput. Methods Programs Biomed..

[49] Kang J., Choi Y.J., Kim I.K., Lee H.S., Kim H., Baik S.H., Kim N.K., Lee K.Y. (2021). LASSO-Based Machine Learning Algorithm for Prediction of Lymph Node Metastasis in T1 Colorectal Cancer. Cancer Res. Treat..

[50] Leitheiser M., Capper D., Seegerer P., Lehmann A., Schüller U., Müller K.R., Klauschen F., Jurmeister P., Bockmayr M. (2022). Machine learning models predict the primary sites of head and neck squamous cell carcinoma metastases based on DNA methylation. J. Pathol..

[51] Wei W., Li Y., Huang T. (2023). Using Machine Learning Methods to Study Colorectal Cancer Tumor Micro-Environment and Its Biomarkers. Int. J. Mol. Sci..

[52] Devi S., Gaikwad S.R., Harikrishnan R. (2023). Prediction and Detection of Cervical Malignancy Using Machine Learning Models. Asian Pac. J. Cancer Prev..

[53] Hochreiter S., Schmidhuber J. (1997). Long Short-Term Memory. Neural Comput..

[54] Bray F., Laversanne M., Sung H., Ferlay J., Siegel R.L., Soerjomataram I., Jemal A. (2024). Global cancer statistics 2022: GLOBOCAN estimates of incidence and mortality worldwide for 36 cancers in 185 countries. CA Cancer J. Clin..

[55] Shi X., Guo S., Duan Q., Zhang W., Gao S., Jing W., Jiang G., Kong X., Li P., Li Y. (2024). Detection and characterization of pancreatic and biliary tract cancers using cell-free DNA fragmentomics. J. Exp. Clin. Cancer Res..

