# Supplementary material for: EM-DeepSD: A Deep Neural Network Model Based on Cell-Free DNA End-Motif Signal Decomposition for Cancer Diagnosis
Source: Diagnostics (Basel). 2025 May 1;15(9):1156. doi: 10.3390/diagnostics15091156 (PMC12071254; doi:10.3390/diagnostics15091156)
Supplement: Supplementary file 1 [file diagnostics-15-01156-s001.zip › Supplementary.pdf]

Supplemental Information

EM-DeepSD: A Deep Neural Network Model Based on Cell-Free DNA End-Motif Signal Decomposition for Cancer Diagnosis

Zhi-Yang Zhao[1a†], Chang-Ling Huang[1b†], Tong-Min Wang[2c], Shi-Hao Zhou[1d], Lu Pei[2e], Wen-Hui Jia[1f], Wei-Hua Jia[1*]

[1] School of Public Health, Sun Yat-sen University, Guangzhou, China.

[2] Sun Yat-sen University Cancer Center, Guangzhou, China.

[†] Zhi-Yang Zhao and Chang-Ling Huang contributed equally to this work.

[a] Zhi-Yang Zhao: zhaozhy63@mail2.sysu.edu.cn

[b] Chang-Ling Huang: huangchling3@mail2.sysu.edu.cn

[c] Tong-Min Wang: wangtm@sysucc.org.cn

[d] Shi-Hao Zhou: zhoushh33@mail2.sysu.edu.cn

[e] Lu Pei: peilu@mail2.sysu.edu.cn

[f] Wen-Hui Jia: jiawh3@mail2.sysu.edu.cn

* Corresponding author: Wei-Hua Jia: jiawh@sysucc.org.cn

Figure and Table legend

Supplementary Figure:

Figure S1. Boxplots show the singular value contribution rate of SSA and the Permutation Entropy of EMD for the EMs profile of 268 cfDNA samples.

Figure S2. Differences in EMs between cancer and control group cfDNA for SSA1 and EMD2.

Figure S3. Differences in EMs between cancer and control group cfDNA for SSA2.

Figure S4. Differences in EMs between cancer and control group cfDNA for SSA3 and EMD1.

Figure S5. The effects of cancer stages and sex on model performance.

Supplementary Table:

Table S1. Detailed information of all datasets.

Table S2. Correlation analysis of EMs after alignment with Bowtie2 and Bismark in the WGS dataset

Table S3. 10-fold cross-validation for model hyperparameters selection

Table S4. Classification metrics of MDS-SDs and baseline methods in diagnosis of cancer.

Table S5. Classification metrics of ML models in diagnosis of cancer.

Table S6. In Test Set-1, the AUC values of EM-DeepSSA across different cancer types based on 1000 Bootstrap iterations.

Table S7. Classification metrics for the ablation study of EM-DeepSSA.

Table S8. Classification metrics for the varying window lengths of EM-DeepSSA.

Table S9. Overview of liquid biopsy assays for cancer early detection described in recent publications

Table S10. Summary table of clinical information

Table S11. literature summary table

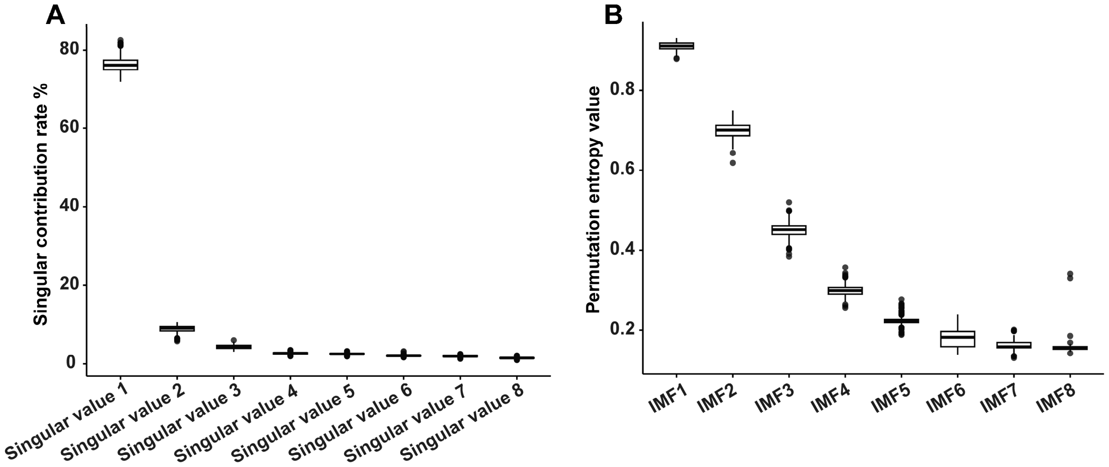

Figure S1. Boxplots show the singular value contribution rate of SSA (**A**) and the Permutation Entropy of EMD (**B**)for the EMs profile of 268 cfDNA samples.

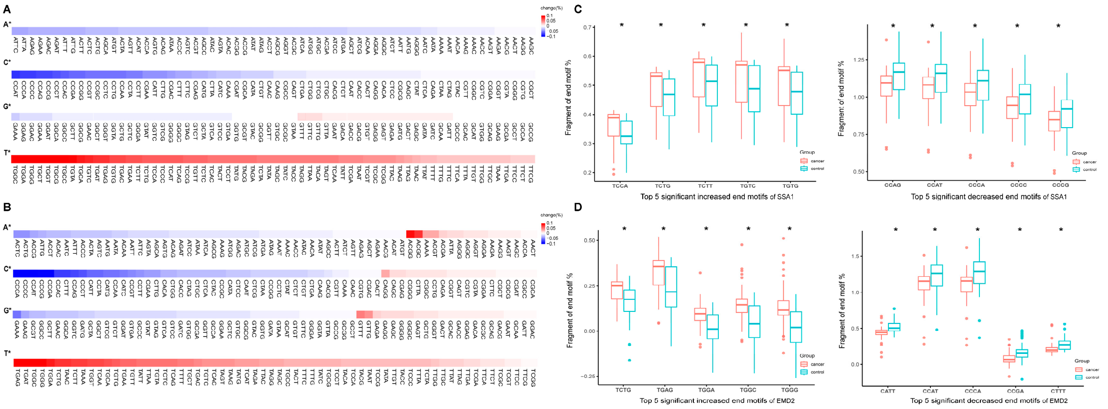

Figure S2. Differences in EMs between cancer and control group cfDNA for SSA1 and EMD2. (A, B) Heatmaps show the changes in EMs profiles of SSA1 (A) and EMD2 (B) in cancer (n=64) compared to control group (n=65). (C, D) Box plots show the top 10 motifs with significant differences in SSA1 (C) and EMD2 (D) between cancer (red) and control group (green). *Wilcoxon rank-sum test with Bonferroni-adjusted p-value <0.05.

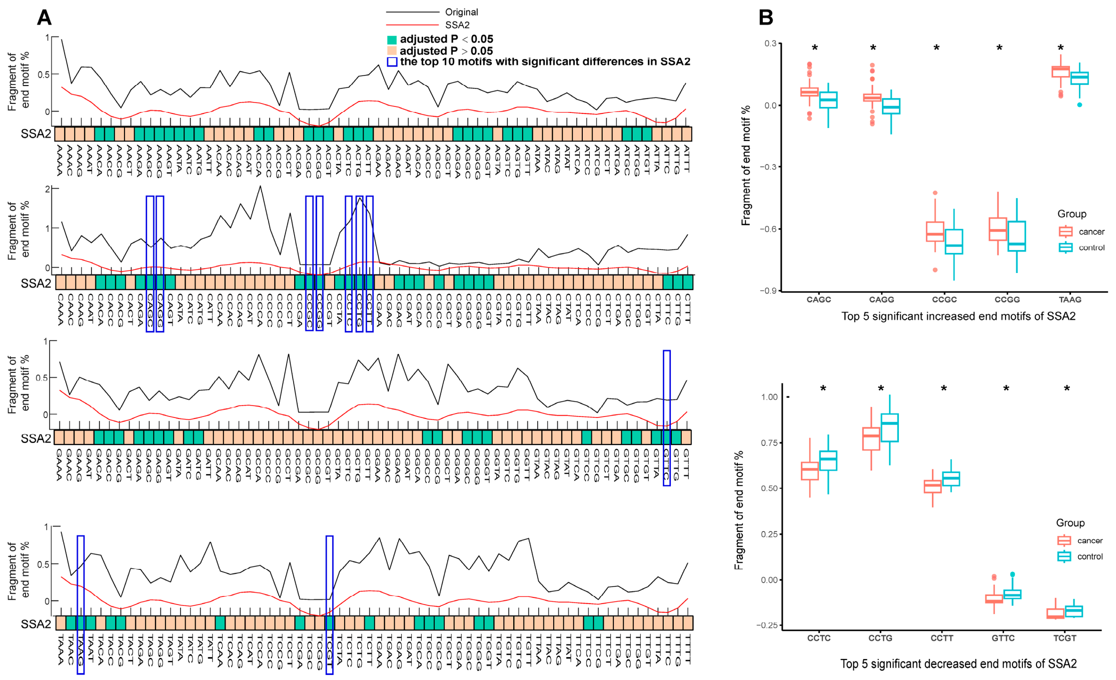

Figure S3. Differences in EMs between cancer and control group cfDNA for SSA2. (A) Plots of the original EM profile and the SSA2 sequence, along with a heatmap that depicts the changes in EMs profiles of SSA2 in cancer (n=64) compared to the control group (n=65). Black lines represent original EMs profile, red lines depict SSA2, green squares represent EMs with significance differences, the top 10 motifs with significant differences in SSA2 are enclosed in blue frames. (B) Box plots show the top 10 motifs with significant differences in frequency between cancer (red) and control group (green). *Wilcoxon rank-sum test with Bonferroni-adjusted p-value <0.05.

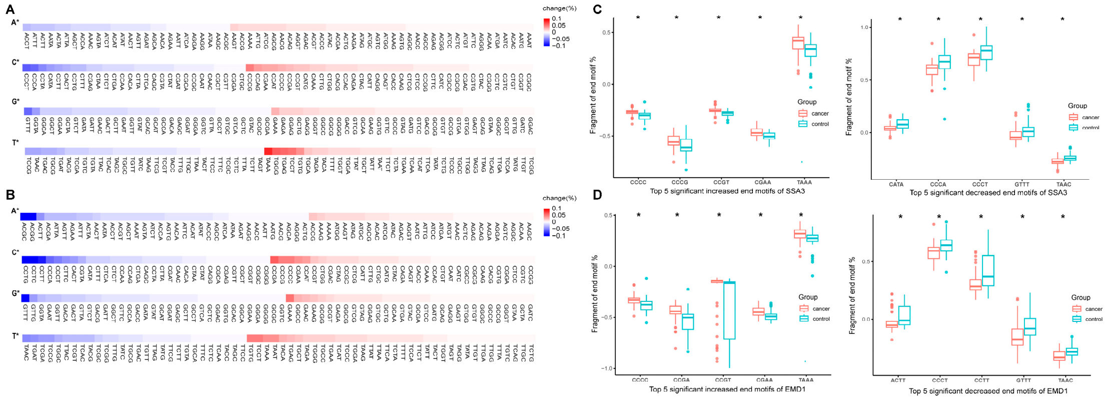

Figure S4. Differences in EMs between cancer and control group cfDNA for SSA3 and EMD1. (A, B) Heatmaps show the changes in EMs profiles of SSA3 (A) and EMD1 (B) in cancer (n=64) compared to control group (n=65). (C, D) Box plots show the top 10 motifs with significant differences in SSA3 (C) and EMD1 (D) between cancer (red) and control group (green). *Wilcoxon rank-sum test with Bonferroni-adjusted p-value <0.05

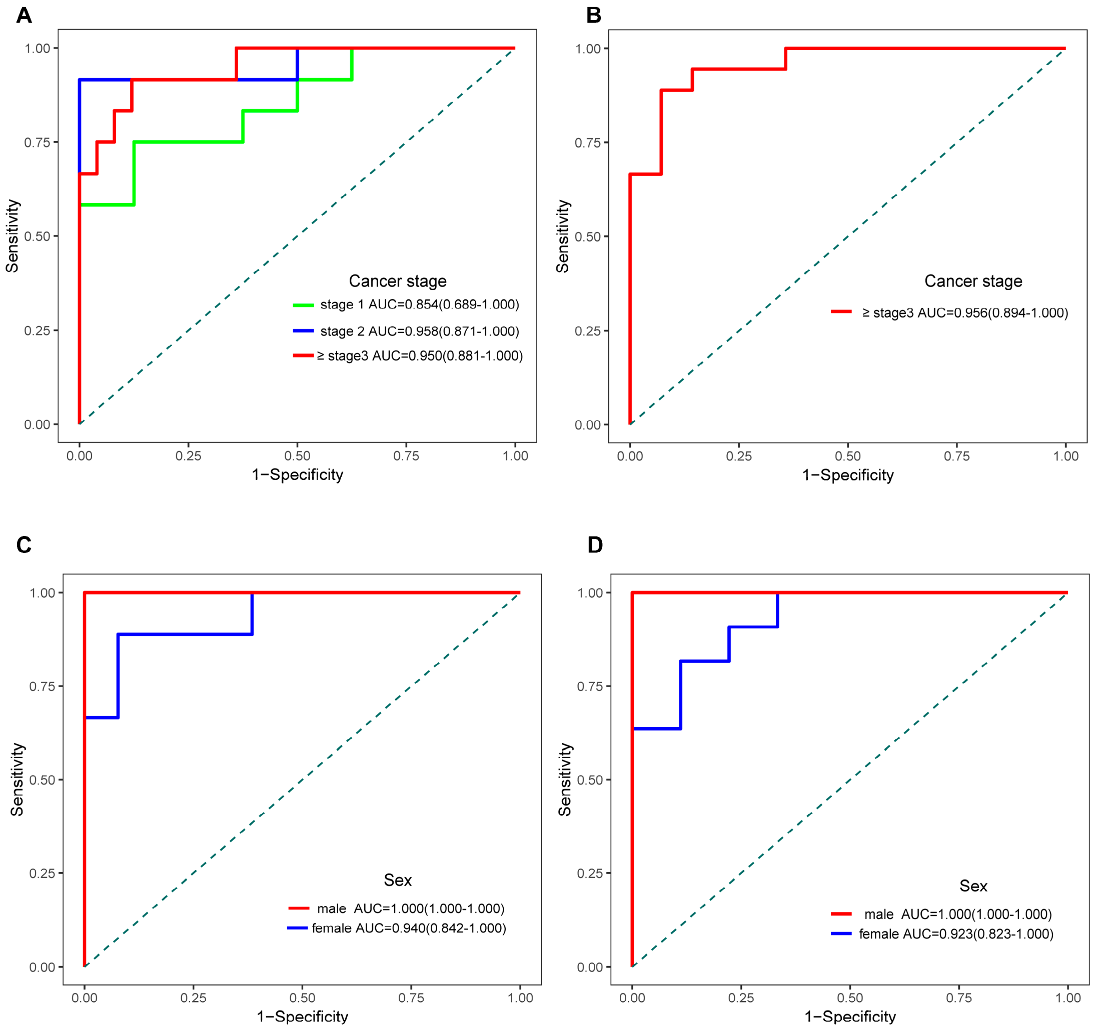

Figure S5. The effects of cancer stages and sex on model performance. (A, B) Receiver operating characteristic (ROC) curves show the detection performance of the EM-DeepSSA model on cancer patients with different stages (I, II, III and above) in the test set 1 (A) and test set 2 (B). (C, D) ROC curves show the detection performance of the EM-DeepSSA model on cancer patients with different sex in the test set 1 (C) and test set 2 (D).
